# Antioxidant and Anti-inflammatory Properties of the Two Varieties of Musa acuminata: An In Vitro Study

**DOI:** 10.7759/cureus.51260

**Published:** 2023-12-28

**Authors:** Balajee V, Lokesh Kumar S, Rajesh Kumar S

**Affiliations:** 1 Department of Oral Medicine, Radiology, and Special Care Dentistry, Saveetha Dental College and Hospitals, Saveetha Institute of Medical and Technical Sciences (SIMATS) Saveetha University, Chennai, IND; 2 Department of Pharmacology, Saveetha Dental College and Hospitals, Saveetha Institute of Medical and Technical Sciences (SIMATS) Saveetha University, Chennai, IND

**Keywords:** affordable medicines, anti-inflammatory agents, musa, musa acuminata, banana, antioxidant activity, antioxidants

## Abstract

Background

Free radicals are involved in the process of carcinogenesis. Conventional antioxidants and anti-inflammatory drugs have the disadvantages of side effects and high costs. Banana peel contains phenolic and non-phenolic antioxidants that are pivotal in removing inflammatory components by inhibiting reactive oxygen species (ROS), protecting protease inhibitors from oxidative damage, and preventing fibroblast degradation which protects the body against the ill effects of free radicals.

Aim and objectives

The present study aimed to evaluate the potential antioxidant and anti-inflammatory activity of peel extracts of the *Musa acuminata *Red Dacca(red banana) and *Musa acuminata *Colla (rasthali).

Materials and methods

The procured unripe peels of red bananas and rasthali bananas were dried, ground into powder, and used to create aqueous and alcoholic extracts. The aqueous extract was made by dissolving 5 grams of peel powder in 25 ml of distilled water, while the alcoholic extract was prepared by heating ethanol to 100°C for 30 minutes. The extracts were combined, shaken for 24 hours, filtered, and stored at 4°C. Following extract preparation, 2,2-diphenyl-1-picrylhydrazyl (DPPH) assay, hydrogen peroxide (H_2_O_2_) assay, bovine serum albumin (BSA) denaturation assay, and egg albumin (EA) denaturation assay were performed to evaluate the antioxidant and anti-inflammatory properties. The assays were performed in varying concentrations for the prepared extracts of red banana and rasthali and the 1:1 ratio combination extract of both varieties. The obtained data were tabulated and statistically tested using the IBM SPSS Statistics for Windows, Version 22.0 (Released 2013; IBM Corp., Armonk, New York, United States) by the Kruskal-Wallis test with the statistical significance set at p≤0.05.

Results

Results highlighted variations in the antioxidant and anti-inflammatory properties of the banana peel extracts and the standard used in all the assays, but there was no statistically significant difference between the extracts and the standard (p>0.05). There was an increase in the antioxidant and anti-inflammatory activity with an increase in the concentration of both the extracts and the standard. The 1:1 ratio combination extract showed the highest antioxidant property among the banana extracts in the majority of the concentrations in the DPPH assay, whereas the rasthali extract showed the same even more than the standard in the H_2_O_2 _assay. The rasthali extract showed the highest anti-inflammatory property in all the concentrations in the BSA assay, and the 1:1 ratio combination extract showed the same in the EA assay.

Conclusion

The banana peel extracts showed comparable antioxidant and anti-inflammatory properties with that of the standard in all the assays with no statistically significant difference. There was a rising trend in the properties with an increase in their concentration. Red banana and rasthali peel extracts, either individually or in combination, could be a promising, effective, and cost-effective alternative or adjunct to the currently available antioxidant medications.

## Introduction

The name "banana" refers to the two subgroups of farmed species of the genus *Musa*: sweet bananas and plantains. The Musaceae family consists of three genera: *Musa*, *Ensete*, and *Musella*. The *Musa* genus contains 65 species of wild and domesticated bananas and plantains. The Ramayana (2000 BC), Arthashastra (250 BC), and Cilappatikāram (500 AD) all have descriptions of the banana, indicating the fruit's historical significance and widespread use in India [1]. Beyond its undeniable gastronomic appeal, recent scientific investigations have unveiled a promising dimension of these fruits: their potential health benefits [2]. In the wake of increasing interest in preventive and therapeutic nutrition, the exploration of bioactive compounds of *Musa acuminata*, particularly its antioxidant and anti-inflammatory properties, has assumed paramount significance [3]. Bananas are a treasure trove of antioxidant components, with vitamin C and vitamin A. Vitamin C, a well-known antioxidant, plays a pivotal role in neutralizing free radicals that cause oxidative stress. Additionally, bananas contain an array of phytochemicals, such as flavonoids and polyphenols, which have demonstrated potent antioxidant effects. These compounds act as molecular shields, safeguarding our cells from damage caused by reactive oxygen species (ROS) [4]. Bananas contain anti-inflammatory components that could have a significant impact on human health. One such component is bromelain, an enzyme known for its anti-inflammatory properties [5]. Bromelain has been studied for its potential to reduce inflammation and alleviate symptoms in conditions such as osteoarthritis. Furthermore, bananas are a rich source of vitamin B6, which is involved in the synthesis of anti-inflammatory molecules within the body. These combined components suggest that bananas may possess a dual-action approach to combatting inflammation, making them a potential ally in the quest for better health [6].

Antioxidants are the molecular components that play a major role in maintaining cellular health. Oxidative stress, often induced by an imbalance between free radicals and antioxidants in the body, has been implicated in numerous chronic diseases, including cancer, cardiovascular ailments, and neurodegenerative disorders. The rich tapestry of bioactive compounds found in *Musa acuminata* suggests that it could be a promising source of antioxidants, capable of mitigating the destructive effects of oxidative stress. Thus, it becomes imperative to subject these fruits to a vigorous in vitro evaluation, where controlled conditions enable precise evaluation of their antioxidant potential [7]. Despite substantial progress in cancer screening, diagnosis, and therapy, there is an urgent need for more potent anticancer drugs with enhanced safety profiles to ultimately eliminate this devastating disease [8].

Banana peels are a natural source of antioxidants and phytochemicals that can counteract harmful free radicals. These include flavonoids like leucocyanidin and quercetin as well as secondary metabolites such as saponins, tannins, alkaloids, and phenols, all of which are found in unripe banana plantain pulp. These compounds give banana peels and pulp robust antioxidant properties and antibacterial peptide activity. The flavonoids present in bananas are particularly effective at reducing harmful hydroperoxides and conjugated dienes by activating enzymes like superoxide dismutase (SOD) and catalase [9]. Additionally, banana peels contain non-phenolic antioxidants like ascorbic acid, beta-carotene, and cyanidin, along with sterols such as stigmasterol, sitosterol, and campesterol. Notably, banana peels are rich in carotenoids, specifically trans-beta-carotene, trans-alfa-carotene, and cis-beta-carotene, each present in specific quantities per unit of dry weight. Moreover, stigmasterol can inhibit the production of inflammatory markers like TNF-a, IL-6, IL-1b, iNOS, and COX-2 while promoting the expression of the anti-inflammatory mediator IL-10. These antioxidants play a pivotal role in eliminating byproducts of inflammation, protecting protease inhibitors from oxidative damage, and preventing the damage inflicted on cells like fibroblasts by ROS [10]. Ethyl acetate sub-fraction of the ethanol extract of banana (*Musa paradisiaca*) soft piths (BSPs) has proven to exhibit potent cytotoxic and anti-proliferative activity against the human oral squamous cell carcinoma (OSCC) cell line (HSC-4) [11].

The emergence of drug resistance and the challenge of precisely targeting cancerous cells represent the two primary hurdles in cancer treatment. Despite significant advancements in cancer management, there remains a pressing need for more effective and safer medications. Also, some antioxidants like lycopene have side effects like increased risk of bleeding tendency, atherosclerosis, and myocardial infarction [12]. Hence, this study is an attempt to overcome all such side effects and provide an efficient natural antioxidant and anti-inflammatory alternative to presently available medications. This study aimed at a meticulous in vitro journey, designed to evaluate the antioxidant potential within two distinct varieties of *Musa acuminata.*

The findings of this research work were previously presented as a poster at the 2023 Saveetha Transdisciplinary Annual Research (STAR) Summit held on June 15, 2023, in Chennai, India.

## Materials and methods

The present study has been waived for ethical clearance by the Institutional Human Ethical Committee, Saveetha Dental College and Hospitals, Chennai, India (reference number: IHEC/SDC/WAIVER CERT-2205/23/03) before data collection. The in vitro* *investigations were conducted in the Gold Lab, Saveetha Dental College and Hospitals, Chennai, India. The red banana and rasthali fruits were procured from a local fruit and vegetable market in Chennai, India.

Preparation of extract

**Figure 1:**
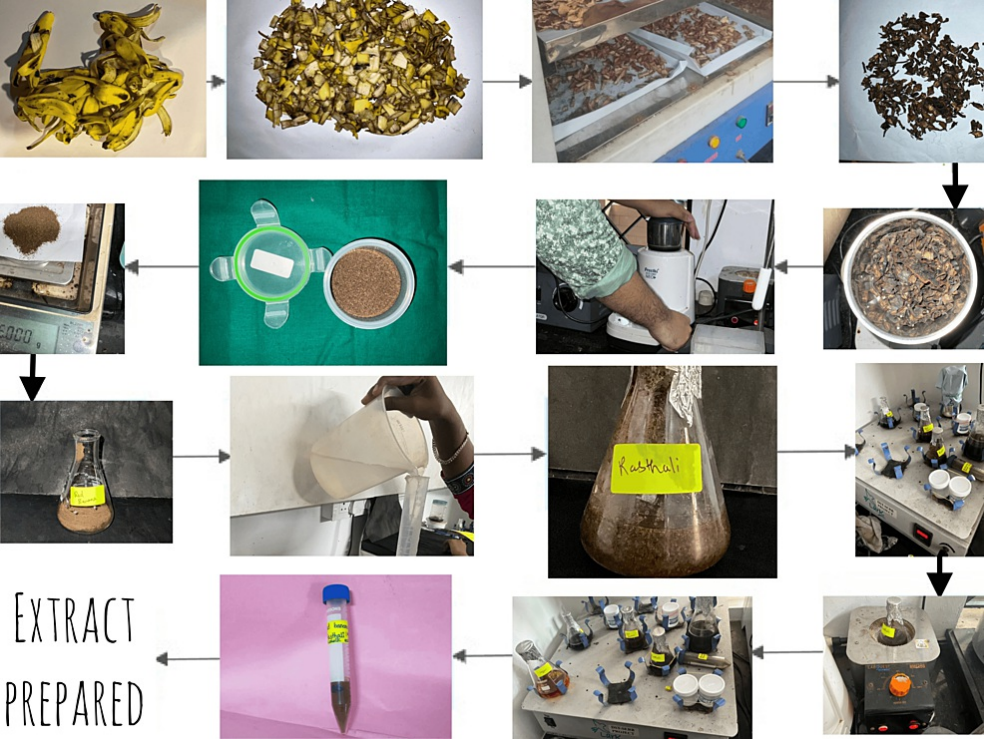
Preparation of banana peel extract

Before the peel was prepared for aqueous and alcoholic extracts, the unripe banana fruit of *Musa acuminata *(red banana and rasthali) was separated, cleaned, and dried. After being maintained for 48 hours at 40° centigrade in a hot air oven, the peels were ground into powder. Five grams of dried peel powder from unripe red bananas and rasthali were dissolved in 25 ml of distilled water to create the aqueous extract. A similar process was used to create the alcoholic extract. Alcoholic extract was produced by heating ethanol, an organic solvent, to a temperature of 100°C for 30 minutes. The two extracts were then combined to produce aqueous alcoholic extract. Cotton plugs were inserted on top of the conical flasks holding the extract to stop evaporation. In an orbital shaker, the extract was shaken for 24 hours at 250 revolutions per minute (rpm). They were filtered twice once with muslin cloths and once with filter paper after being shaken all night. The resultant extracts were stored at 4°C. The preparation of the extract is depicted in Figure 1.

The extracts of rasthali, red banana, and their 1:1 ratio combination were tested for their antioxidant and anti-inflammatory properties.

Antioxidant activity

The *Musa acuminata* extracts were tested for antioxidant activity using two assays, namely, the 2,2-diphenyl-1-picrylhydrazyl (DPPH) free radical assay and hydrogen peroxide (H_2_O_2_) assay.

DPPH Free Radical Scavenging Assay

DPPH was prepared in methanol, and a fresh working solution was prepared by diluting the stock solution to a final concentration of 20 µM in methanol for each assay. Different concentrations (10, 20, 30, 40, and 50 µg/mL) of the *Musa acuminata* extract were added to 200 µL of the DPPH working solution in a 96-well plate [13]. The plate was incubated in the dark for 30 minutes at room temperature. The absorbance was measured at 517 nm using a microplate reader. Methanol was used as a blank. The percentage of DPPH scavenging activity was calculated using the formula DPPH Free Radical Scavenging Activity (%)=[(Acontrol-Asample)/Acontrol]×100, where Acontrol is the absorbance of the control (DPPH solution without the sample) and Asample is the absorbance of the sample (DPPH solution with the *Musa acuminata* extract). The positive control group consisted of ascorbic acid (1 mg/mL).

H_2_O_2_ Assay

Hydroxyl radical scavenging assay was used in this study to evaluate the antioxidant activity using the method proposed by Halliwell et al. 1 mL of reaction mixture with 100 µL of 28 mM of 2-deoxy-2-ribose was prepared. To that, various concentrations of *Musa acuminata *extracts (10-50 µg/mL) were added. Along with that, 200 µL of 200 µm ferric chloride, 200 µL of ethylenediaminetetraacetic acid (EDTA), and 100 µL of ascorbic acid were added. Then it was incubated for one hour at 37°C, and the optical density was measured at 532 nm against the blank solution. Vitamin E was used as a positive control [14]. The formula hydroxyl radical scavenging activity (%)=[(Ablank-Asample)/Ablank]×100 was used, where Ablank is the absorbance of the control reaction (without sample) and Asample is the absorbance of the reaction with the sample.

Anti-inflammatory activity

The *Musa acuminata *extracts were tested for anti-inflammatory activity using two assays, namely, the bovine serum albumin (BSA) denaturation assay and egg albumin (EA) denaturation assay.

BSA Denaturation Assay

0.45 mL of BSA was mixed with 0.05 mL of different concentrations (10-50 µg/mL) of *Musa acuminata* extracts. The pH was adjusted to 6.3. Then it was kept at room temperature for 10 minutes and incubated in the water bath at 55°C for 30 minutes. Diclofenac sodium was used as the standard group, while dimethyl sulfoxide was used as control. Then, the samples were measured spectrophotometrically at 660 nm [15]. The percentage of protein denaturation was determined utilizing the following equation: % inhibition=Absorbance of control-Absorbance of sample×100/Absorbance of control.

EA Denaturation Assay

An EA denaturation assay was performed by mixing 0.2 mL of fresh EA with 2.8 mL of phosphate buffer. Different concentrations (10-50 µg/mL) of *Musa acuminata* extracts were added to the reaction mixture. The pH was adjusted to 6.3. Then it was kept at room temperature for 10 minutes and incubated in the water bath at 55°C for 30 minutes. Diclofenac sodium was used as the standard group, while dimethyl sulfoxide was used as control [16]. Then, the samples were measured spectrophotometrically at 660 nm. The percentage of protein denaturation was determined utilizing the following equation: % inhibition=Absorbance of control-Absorbance of sample×100/Absorbance of control.

Statistical analysis

The data generated were subjected to statistical analysis through the IBM SPSS Statistics for Windows, Version 22.0 (Released 2013; IBM Corp., Armonk, New York, United States). The Kruskal-Wallis test was performed with a significance level set at p≤0.05.

## Results

DPPH free radical scavenging assay

**Figure 2:**
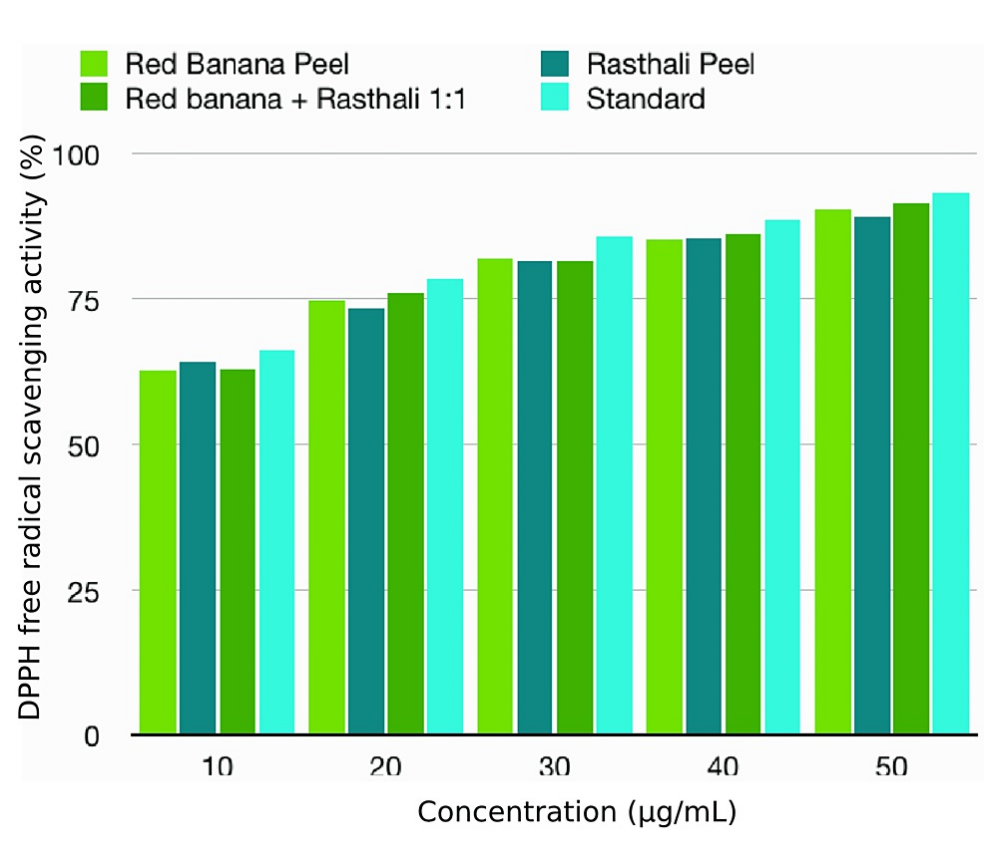
Antioxidant properties of banana peel extracts by DPPH assay DPPH: 2,2-diphenyl-1-picrylhydrazyl

DPPH free radical scavenging assay showed that the antioxidant activity of the banana peel extracts under investigation had comparable results with the standard which had the highest antioxidant activity in all tested concentrations. The rasthali extract had the highest antioxidant capacity (64.31%) at a concentration of 10 µg/mL among the banana varieties. The 1:1 ratio combination extract had higher antioxidant property (75.97%, 86.31%, and 86.31%) than red banana and rasthali in the concentrations of 20, 40, and 50 µg/mL, respectively. Nevertheless, the red banana extract showed a higher antioxidant property (82.05%) than the other varieties at the concentration of 30 µg/mL. There was a rise in the antioxidant activity with the increase in concentration in both the standard and the extracts. However, there was no statistically significant difference (p>0.05) between the tested extracts and standard in all the concentrations. The results of the DPPH free radical scavenging assay are shown in Table 1 and Figure 2. 

**Table 1:** Antioxidant properties of the banana peel extracts by DPPH free radical scavenging assay DPPH: 2,2-diphenyl-1-picrylhydrazyl; %: percentage The values are expressed in % of DPPH free radical scavenging activity. The statistical test used was the Kruskal-Wallis test. P≤0.05 was considered statistically significant

Test	Concentration (µg/ml)	Red banana peel (%)	Rasthali peel (%)	Red banana+rasthali peel (1:1) (%)	Standard (%)	p-value
DPPH assay	10	62.71	64.31	63.04	66.25	0.392
	20	74.60	73.52	75.97	78.52	0.392
	30	82.05	81.54	81.55	85.63	0.392
	40	85.28	85.49	86.31	88.68	0.392
	50	90.55	89.18	91.50	93.15	0.392

H_2_O_2_ assay

**Figure 3:**
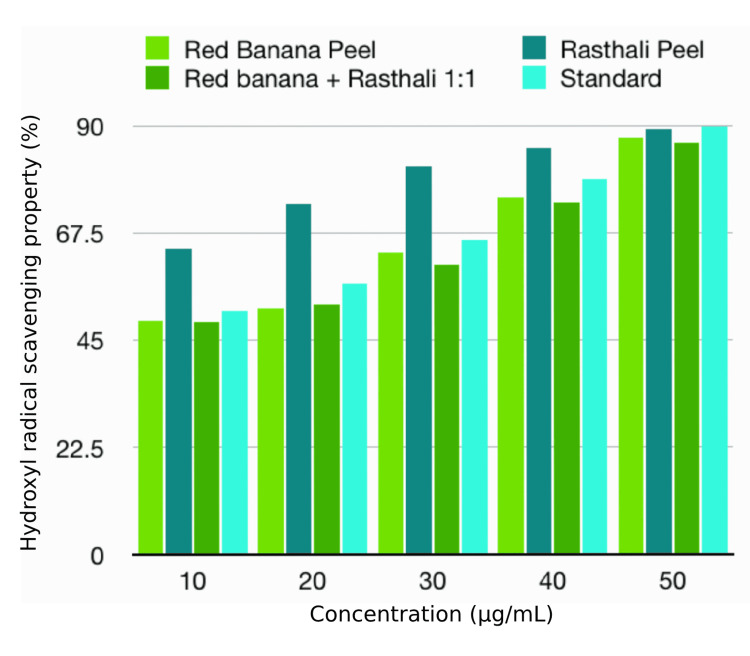
Antioxidant properties of banana peel extracts by H2O2 assay H_2_O_2_: hydrogen peroxide

H_2_O_2_ assay showed that the antioxidant activity of the banana peel extracts under investigation ranged from 48.76% to 89.9%. The rasthali peel extract showed the highest antioxidant property (64.31%, 73.52%, 81.54%, and 85.49%) even above the standard in the concentrations of 10, 20, 30, and 40 µg/mL, respectively. However, the rasthali extract showed a high antioxidant property (89.18%) compared to the other varieties but less than the standard in the concentration of 50 µg/mL. There was a rise in the antioxidant activity with the increase in concentration in both the standard and the extracts. However, there was no statistically significant difference (p>0.05) between the tested extracts and standard in all the concentrations. The antioxidant properties of the different banana peel extracts, as compared to the standard, by H_2_O_2_ assay are shown in Table 2 and Figure 3. 

**Table 2:** Antioxidant properties of the banana peel extracts by H2O2 assay H_2_O_2_: hydrogen peroxide; %: percentage The values are expressed in % of hydroxyl radical scavenging activity. The statistical test used was the Kruskal-Wallis test. P≤0.05 was considered statistically significant

Test	Concentration (µg/ml)	Red banana peel (%)	Rasthali peel (%)	Red banana+rasthali peel (1:1) (%)	Standard (%)	p-value
H_2_O_2_ assay	10	49.11	64.31	48.76	51.10	0.392
	20	51.64	73.52	52.48	56.90	0.368
	30	63.45	81.54	60.90	66.10	0.392
	40	74.97	85.49	73.81	78.80	0.392
	50	87.38	89.18	86.54	89.90	0.392

BSA denaturation assay

**Figure 4:**
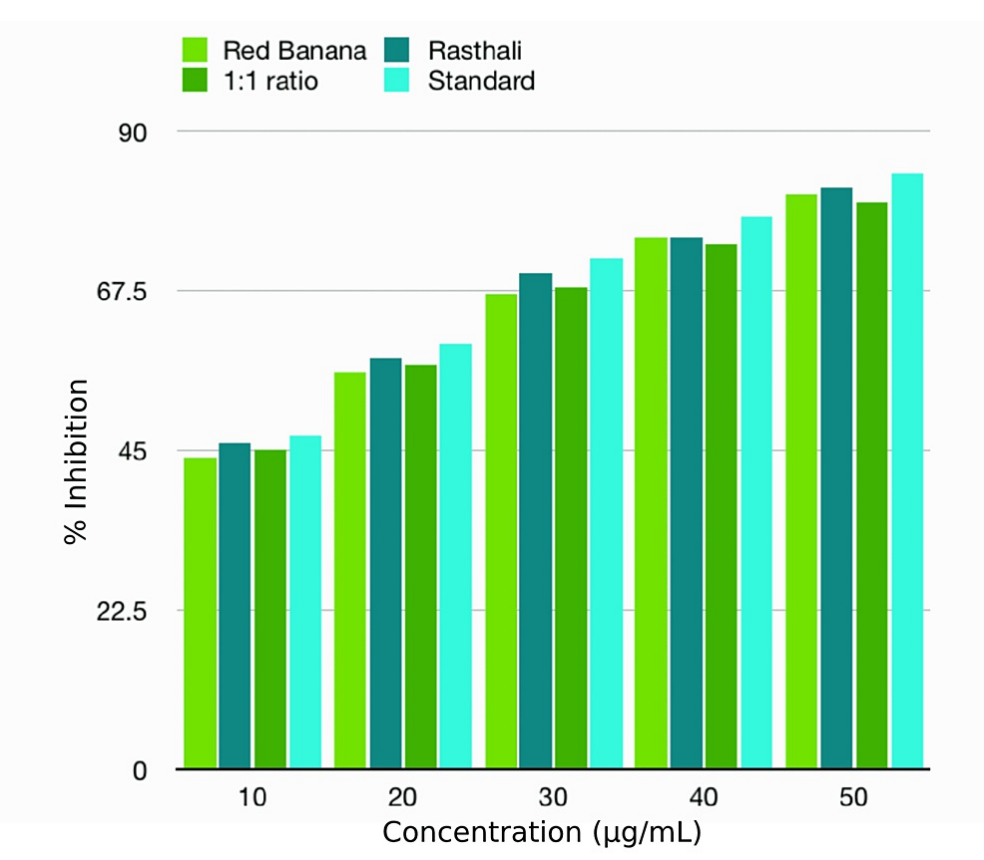
Anti-inflammatory properties of banana peel extracts by BSA denaturation assay BSA: bovine serum albumin

BSA denaturation assay showed that the anti-inflammatory activity of the banana peel extracts and standard under investigation fell between 44% and 84%. The rasthali peel extract showed the highest anti-inflammatory property than the other banana peel extracts in all the tested concentrations. However, the standard still had a higher anti-inflammatory property than the rasthali peel extract. There was a rise in the anti-inflammatory activity with the increase in concentration in both the standard and the extracts. However, there was no statistically significant difference (p>0.05) between the tested extracts and standard in all the concentrations. The anti-inflammatory activities of the banana peel extracts, as compared to the standard, by BSA denaturation assay are depicted in Table 3 and Figure 4. 

**Table 3:** Anti-inflammatory properties of the banana peel extracts by BSA assay BSA: bovine serum albumin; %: percentage The values are expressed in % of inhibition. The statistical test used was the Kruskal-Wallis test. P≤0.05 was considered statistically significant

Test	Concentration (µg/ml)	Red banana peel (%)	Rasthali peel (%)	Red banana+rasthali peel (1:1) (%)	Standard (%)	p-value
BSA assay	10	44	46	45	47	0.392
	20	56	58	57	60	0.392
	30	67	70	68	72	0.392
	40	75	75	74	78	0.392
	50	81	82	80	84	0.392

EA denaturation assay

**Figure 5:**
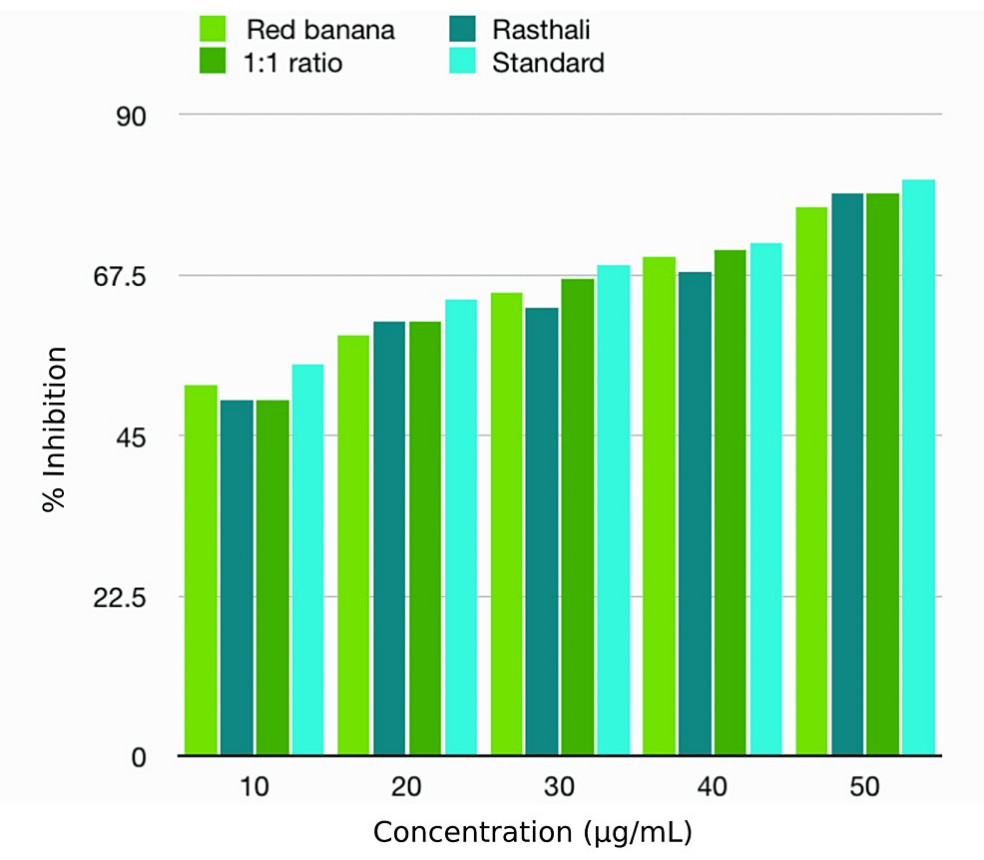
Anti-inflammatory properties of banana peel extracts by EA denaturation assay EA: egg albumin

EA denaturation assay showed that the anti-inflammatory activity of the banana peel extracts and standard under investigation fell between 50% and 81%. The red banana extract (52%) and rasthali extract (61%) showed the highest anti-inflammatory property as compared to that of other banana varieties in the concentrations of 10 and 20 µg/mL, respectively. However, the 1:1 ratio combination extract showed higher anti-inflammatory activity (67%, 71%, and 79%) than the other two individual banana extracts in the concentrations of 30, 40, and 50 µg/mL, respectively. There was a rise in the anti-inflammatory activity with the increase in concentration in both the standard and the extracts. However, there was no statistically significant difference (p>0.05) between the tested extracts and standard in all the concentrations. The anti-inflammatory activities of the banana peel extracts, as compared to the standard, by EA denaturation assay are depicted in Table 4 and Figure 5.

**Table 4:** Anti-inflammatory properties of the banana peel extracts by EA denaturation assay EA: egg albumin; %: percentage The values are expressed in % of inhibition. The statistical test used was the Kruskal-Wallis test. P≤0.05 was considered statistically significant

Test	Concentration (µg/ml)	Red banana peel (%)	Rasthali peel (%)	Red banana+rasthali peel (1:1) (%)	Standard (%)	p-value
EA denaturation assay	10	52	50	50	55	0.392
	20	59	61	61	64	0.392
	30	65	63	67	69	0.392
	40	70	68	71	72	0.392
	50	77	79	79	81	0.392

## Discussion

Banana peels come in various forms and are a natural source of antioxidants and phytochemicals, including compounds that combat free radicals. Flavonoids like leucocyanidin and quercetin have been isolated from unripe banana pulp. Phytochemical analysis reveals secondary metabolites like flavonoids, saponins, tannins, alkaloids, and phenols in banana peel and pulp extracts. These compounds exhibit antioxidant properties and antibacterial effects. Banana peels also contain non-phenolic antioxidants such as ascorbic acid, beta-carotene, and cyanidin. Sterols like stigmasterol, sitosterol, and campesterol, along with tripenic alcohols, are found in banana peels [17]. The active ingredients in banana peels have a wide range of pharmacological applications, including anti-inflammatory and antibacterial properties, that can be used to prevent the onset of various inflammatory disorders.

According to the structure-activity relationship of flavonoids, the presence of functional groups in their nuclear structures is related to their ability to operate as chelators, scavenge free radicals, and act as antioxidants [18]. They also stated that flavonoids' antioxidant and chelating capabilities were responsible for the majority of the health advantages associated with their ingestion. Flavonoids have been demonstrated to have antimutagenic and antitumoral capabilities as a result of these qualities [19].

Additionally, they can block several enzymes, such as prostaglandin synthase, which is essential for the synthesis of eicosanoids. By limiting the activity of hyaluronidase, this inhibition aids in maintaining the integrity of connective tissues and may also prevent the spread of pathogens or tumor metastases [20]. Additionally, it is known that flavonoids preferentially suffer oxidation, which can prevent the oxidation of the body's natural water-soluble antioxidants such as ascorbic acid [21].

Antioxidants, like stigmasterol, play a role in inhibiting the production of inflammatory molecules and protecting against oxidative damage. Antioxidants are vital for eliminating inflammatory products by combating proteases and ROS. They also shield protease inhibitors from oxidative harm and prevent ROS-induced damage to cells like fibroblasts [22]. In the current study, the methanolic rasthali peel extract had more antioxidant activity as compared to that of the methanolic red banana peel extract in the H_2_O_2_ assay which is in accordance with the study conducted by Siji and Nandini in 2017 [23]. Nevertheless, the 1:1 ratio extract combination of both rasthali and red banana showed superior antioxidant properties than the individual extracts in the concentrations of 20, 40, and 80 µg/mL during the DPPH assay. In the current study, the rasthali peel extract also had more anti-inflammatory activity as compared to that of the red banana peel extract which is in agreement with the study conducted by Arora et al. in 2008 [24]. The banana peel extracts showed comparable antioxidant and anti-inflammatory properties with that of standard in all concentrations tested in all the assays. It is noteworthy that the rasthali peel extract showed a superior antioxidant property than the standard in the concentrations of 10, 20, 30, and 40 µg/mL in the H_2_O_2_ assay. However, there was no statistically significant difference in the antioxidant and anti-inflammatory properties between the two banana peel extracts and their 1:1 ratio combination. All the assays seemed to show a rising trend in the antioxidant and anti-inflammatory properties with an increase in the concentration of both the standard and the banana peel extracts.

In 2011, Baskar et al.investigated the phytochemical content and antioxidant potential of nine different local varieties of banana peels. As a result of its high 2,2'-azino-bis(3-ethylbenzthiazoline-6-sulfonic acid) (ABTS) scavenging activity, rasthali was found to have a higher total phenol content than the other species. The higher flavonoid content of poovan bananas, on the other hand, is closely linked to their capacity to prevent lipid peroxidation. It was concluded that the banana peel extracts from these varieties may be useful in the fight against diseases brought on by free radicals because of the link between the phytochemical content of banana peels and their ability to scavenge free radicals [25].

Sharma et al. assessed the comparative antioxidant activity of *Musa acuminata *and *Musa paradisiaca *by electron transfer assay, total phenolic content, and DPPH free radical scavenging assay. The results of this investigation showed that the methanolic extract of the peel of the ripe fruit of *Musa acuminata *showed the maximum phenolic content (1162 mg QE/g of extract) and the pulp of the unripe fruit of *Musa acuminata* exhibited the maximum antioxidant properties, i.e., 85.38% inhibition against free radicals, while the acetone extract of the peel of unripe banana fruit from *Musa paradisiaca* demonstrated the maximum antioxidant property with 73.61% inhibition, which is still less than that of *Musa acuminata*. The ethanolic extract from the ripe pulp of *Musa acuminata *and the methanolic extract from the ripe peel of *Musa paradisiaca *showed the maximum reducing power [7].

According to Kaimal et al., animals that are hyperlipidemic and experiencing oxidative stress may benefit from the antioxidant and hypolipidemic effects of an ethanolic extract of *Musa AAA* (Chenkadali/red banana) fruits. The ethanolic fruit extract of *Musa AAA *significantly decreased the levels of cholesterol, triacylglycerol, alanine transferase activity, and lipid peroxides. It also significantly increased the reduced glutathione (GSH) in the liver and pancreas. The activity was dose-dependent, and though there was no statistically significant difference, the administration of 500 mg/kg body weight of the extract demonstrated high GSH and lower lipid peroxides in the pancreas compared to glibenclamide. It demonstrates that the active ingredients in the lower dose are present in suitable concentrations and worked in concert to lower the levels of tissue lipid peroxides and serum lipids. Consequently, it might not always be advantageous to raise the dosage to increase the concentration of active ingredients in the fruit extract to bring about the intended biological effect, in the event that a high concentration of any component has the opposite effect [26].

This study has demonstrated that the banana peel can act as an effective natural antioxidant against free radicals. Ascorbic acid, beta-carotene, and dopamine [27] are a few of the many bioactive compounds that are abundant in bananas and have health benefits like strong antioxidant and free radical scavenging properties [28]. Together, these antioxidant substances and antioxidant activity lower the risk of degenerative illnesses including cancer, cardiovascular disease, etc. Banana peel also contains anti-inflammatory agents such as trigonelline which inhibits bacterial enzymes and nucleic acid synthesis, isovanillic acid which suppresses TNF-α production, and ferulic acid which inhibits the production of pro-inflammatory signaling and cytokines.

While banana peels contain several beneficial substances, studies on their nutritional, pharmaceutical, and bioactive potential have been limited. Ripe and unripe banana peels contain fiber, pectin, phytosterols, phenolics, biogenic amines, and carotenoids. The latter four possess antioxidant properties, which are associated with health benefits. However, there's still a lack of conclusive evidence linking banana peels to anti-inflammatory properties. The active ingredients in banana peels have a wide range of pharmacological applications, including anti-inflammatory and antibacterial properties, that can be used to prevent the onset of various inflammatory disorders. To identify the processes underlying the antioxidant potential of these byproduct extracts, additional research is also needed to isolate and characterize the particular phenolic chemicals found in different extracts. The antioxidant properties of these banana varieties can also be compared with the antioxidant properties of lycopene. Being an in vitro investigation, the findings of the research should be confirmed by conducting clinical trials to know the clinical efficacy of red banana and rasthali extracts.

## Conclusions

The present study demonstrated comparable antioxidant and anti-inflammatory properties of peel extracts of the rasthali, red banana, and their 1:1 ratio combination with that of the standard used. There was no statistical difference in the antioxidant and anti-inflammatory properties of the tested peel extracts and standards in all concentrations. There was a rising trend in the tested properties with an increase in the concentration. Hence, the rasthali and red banana peel extracts can be promising and cost-effective alternatives or adjuncts to the synthetic anti-inflammatory drugs that are currently available. Clinical trials are warranted to evaluate the clinical efficacy of the banana peel extracts in different forms.
